# Bacteriocin as Weapons in the Marine Animal-Associated Bacteria Warfare: Inventory and Potential Applications as an Aquaculture Probiotic

**DOI:** 10.3390/md8041153

**Published:** 2010-04-04

**Authors:** Florie Desriac, Diane Defer, Nathalie Bourgougnon, Benjamin Brillet, Patrick Le Chevalier, Yannick Fleury

**Affiliations:** 1 Université Européenne de Bretagne, Université de Brest, Institut Universitaire de Technologie, Laboratoire, Universitaire de Biodiversité et d’Ecologie Microbienne EA3882, 6 Rue de l’Université, 29334 Quimper Cedex, France; E-Mails: floriedesriac@hotmail.fr (F.D.); benjamin.brillet@univ-brest.fr (B.B.); patrick.lechevalier@univ-brest.fr (P.L.C.); 2 Université Européenne de Bretagne, Université de Bretagne Sud, Centre de Recherche Saint Maudé, Laboratoire de Biotechnologie et Chimie Marines EA3884, 56321 Lorient Cedex, France; E-Mails: diane.defer@univ-ubs.fr (D.D.); nathalie.bourgougnon@univ-ubs.fr (N.B.)

**Keywords:** aquaculture, BLIS, bacteriocin, probiotic

## Abstract

As the association of marine animals with bacteria has become more commonly recognized, researchers have increasingly questioned whether these animals actually produce many of the bioactive compounds originally isolated from them. Bacteriocins, ribosomally synthesized antibiotic peptides, constitute one of the most potent weapons to fight against pathogen infections. Indeed, bacteriocinogenic bacteria may prevent pathogen dissemination by occupying the same ecological niche. Bacteriocinogenic strains associated with marine animals are a relevant source for isolation of probiotics. This review draws up an inventory of the marine bacteriocinogenic strains isolated from animal-associated microbial communities, known to date. Bacteriocin-like inhibitory substances (BLIS) and fully-characterized bacteriocins are described. Finally, their applications as probiotics in aquaculture are discussed.

## 1. Introduction

According to a FAO report, the average consumption of aquaculture products relative to total *per capita* fish for human consumption rose from 14% in 1986 to 47% in 2006 and it can be expected to reach 50% in the next few years. However, the development of aquaculture farming will have to be backed up with appropriately relevant management practices, in particular by decreasing its environmental impact and limiting the associated infectious epizooties. Indeed, as in all animal industries, development and intensification generate higher population densities which exacerbate disease processes, leading to stock mortality [1]. Major economic losses in cultured fish worldwide result from a relatively small number of opportunistic pathogens bacteria [2]. *Vibrio* is one of the most important pathogenic recognized in larval cultures, provoking a high mortality [3,4]. Furthermore, fear of aquaculture farming increases with climate change. Indeed, a recent report has shown that numerous bacteria display greater virulence at higher temperatures due to reduced resistance and increased virulence and transmission [5]. At the same time, use of prophylactic antibiotics is detrimental to aquatic and terrestrial environments, animal and human health [6,7]. That’s why authorities such as the European Authority have chosen to limit antibiotic use as a curative situation. In this context, scientific communities have proposed friendly alternatives such as vaccines [1], antibiotic substitutes [8] or use of probiotic [9]. Bacteriocinogenic bacterial strains appear to be an excellent candidate for a friendly alternative since bacteriocin would be used as an antibiotic substitute [10], whereas bacteria would be a potential probiotic [11].

Bacteriocins are ribosomally synthesized proteinaceous compounds, lethal to bacteria closely related to the producing bacteria [10,12], the latter being protected by an immunity phenomenon. The role of bacteriocins in microbial communities hasn’t been well-established yet. Bacteriocins may serve as anti-competitor compounds enabling an invasion of a strain or species in an established microbial community [13–15] or act as communication molecules in bacterial consortia like biofilms [11]. Nevertheless, using pure bacteriocins is not practical since it has no economic basis. One way to substitute antibiotics smartly and sustainably will be the selection of bacteriocinogenic and anti-pathogenic strains from animal-associated bacterial microorganisms for use as probiotics.

In this review, the first section deals with a definition of probiotics and their mode of action, while the second part is dedicated to bacteriocin knowledge to date. Then an inventory of marine bacteriocin-like inhibitory substances (BLIS) producing bacteria in the literature is drawn up. The last section is about an efficient strategy to select bacteriocinogenic bacteria.

## 2. Probiotics for Aquaculture

In 1908 Elie Metchnikoff started the discipline of probiotics by reporting for the first time dietary supplements containing potentially beneficial micro-organisms. However, Kollath was the first to suggest, in 1953, the term “probiotics” to designate organic or inorganic substances that are essential to a healthy development of life [16]. Two decades later, Parker used the term “probiotic” to describe animal feed supplements that contribute to the gut microbial communities of the host [17]. In 1989, Fuller suggested another definition widely used since: “*A live microbial feed supplement which beneficially affects the host animal by improving its intestinal balance*” [18]. This revised definition differs from Parker’s one by emphasizing the importance of live cells that permit the formal exclusion of antibiotics from the probiotics group. In 1999, Salminen proposed a new definition: “*Probiotics are microbial cell preparations or components of microbial cells that have a beneficial effect on the health and well-being of the host*” [19]. This implies that non-viable forms of probiotics have also been shown to have health effects and should not restrict the utilization of probiotics in food [20]. The International Scientific Association for Probiotics and Prebiotics recently adopted the definition of the World Health Organization: “*Probiotics are live microorganisms which when administrated in adequate amounts confer a health benefit on host*” [21].

Nevertheless, none of these definitions fit with aquaculture since aquatic animals have a much closer relationship with their environment than terrestrial ones. In fact, in seawater, pathogens proliferate independently of the host, so opportunistic organisms can reach a high density around aquatic animals [22]. Furthermore, it is admitted that bacteria present in aquatic environments influence the composition of the gut microbiota, with surrounding bacteria being continuously ingested [23,24]. The intensive interaction between the environment and the farmed aquatic animals implies that the definition of probiotics has to be adapted for aquaculture. Based on this statement, a new definition for probiotics has been proposed: “*A live microbial adjunct which has a beneficial effect on host by modifying the host-associated or ambient microbial community, by ensuring improved use of the feed or enhancing its nutritional value, by enhancing the host response towards disease, or by improving the quality of its ambient environment*” [25].

This confers to aquaculture probiotics a large possibility to affect the host health positively [26] by competitive exclusion [27], by enzymatic contribution to digestion [11,28,29] and by enhancement of the immune response [30,31] or by the production of inhibitory substances [9]. Inhibitory substance production is probably one of the most studied modes of probiotic action.

## 3. Bacteriocins

### 3.1. Bacteriocin story

To go back to the first bacteriocin descriptions amounts to studying the first works concerning bacterial antagonism. Such bacterial antagonism was described by the pioneers of microbiology during the last decades of the 19^th^ century. At that time, the molecular basis of bacterial inhibition was abstruse, so it was difficult to distinguish antagonism due to bacteriocins from that provoked by other compounds such as antibiotics, organic acids or hydrogen peroxide, except on the basis of their spectrum of activity, usually narrower than that of the other ones. Although Cornil and Babès suggested a very narrow antagonism within the genus *Staphyloccoccus* (“le staphylocoque empêche surtout le staphylocoque”) in their 1885 treatise of bacteriology [32], the scientific community acknowledges the Gratia *et al.* findings [33] in 1925 as the first documented bacteriocin activity. Indeed, it was named colicin V by the same team in 1949 [34]and later microcin V [35].

The term bacteriocin did not appear until the fifties [34]. This bacteriocin definition is based on the properties of the colicins, that is to say, a lethal biosynthesis, a very narrow spectrum of activity limited to the same species as the producer bacteria and a receptor-mediated mechanism of action [36]. In those days, during the fifties and sixties, the bacteriocin world was mainly made up of bacteriocins from Gram negative bacteria [37,38]. Three genera of Gram positive bacteria were studied for bacteriocin production: *Bacillus* sp., *Listeria* sp. and *Staphylococcus* sp., but it should be noted that during the first half of the 20th century, two lantibiotics, one of the most famous bacteriocins to date, were described. Indeed, the first observations of nisin activity could be those of Roger *et al.* [39], while subtilin was identified in 1944 from *Bacillus subtilis* [40]. The exotic amino acid sequences of nisin and subtilin were only elucidated in the early seventies [41,42].

**Figure 1:**
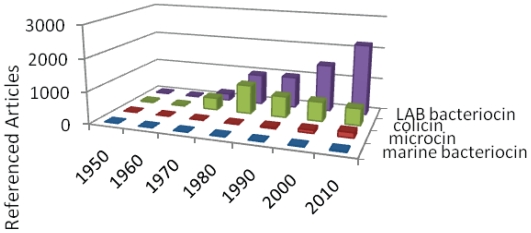
Bacteriocin related publications per 10 years period referenced in Pubmed. The bibliographical data bank, Pubmed, was questioned *per* period of 10 year since 1949. The various keywords employed aimed at distinguishing the various categories of bacteriocins. They were required in title and summary. The different keywords used for query were “Colicin” for colicin, “microcin not colicin” for microcins and “bacteriocin and LAB not colicin not microcin” for LAB bacteriocin.

The eighties saw an increase in the number of publications on bacteriocin for both colicin type- and non colicin bacteriocins (Figure 1). But the attribution of nisin GRAS-status by FDA in 1988 [43] would unleash interest in the bacteriocins produced by lactic acid bacteria. Indeed, the industrial applications and the medical and veterinary potential of these microorganisms considered as technological ones are enormous [44–48]. These bacteriocins have aroused a keen interest which has resulted in an exponential increase in the number of publications, while scientific publications about colicins, which may represent the most extensively studied bacteriocins to date, seem to be stabilizing (Figure 1).

Such interest in LAB bacteriocins has resulted in applications as food preservatives, *eg* antimicrobial ingredients [45–50]. Over the last 20 years, 706 patents based on LAB bacteriocins activity have been recorded around the World, 421 of which were linked to food preservation, and 124 to animal probiotics (http://www.freepatentsonline.com). The non LAB bacteriocins are not devoid of application fields. Applications have also been suggested for plant protection [12,51,52], to prevent local infections in humans [53] and recently in aquaculture [11]. Two dedicated freely available bacteriocin online databases have been assembled: BACTIBASE [54] and BAGEL [55]. Moreover, bacteriocins are part of antimicrobial peptides and on this account, are referenced in various antimicrobial peptide databases such as APD2 [56,57] or CyBase [58].

A new category of bacteriocins has emerged over the last two decades: that of the microcins (Figure 1). These may be considered as the “little sisters” of colicins since they exhibit low molecular weight and are produced by enterobacteriae (for reviews see [35,59,60]). Besides, most microcins exhibit intensive post translational modifications yielding exotic amino acids [61]. In a way, microcins are counterparts of lantibiotics in Gram negative bacteria [61].

Only a few publications are dedicated to bacteriocin production by marine bacteria. Only a few BLIS have been described from marine bacteria and a unique bacteriocin has been fully characterized (see below). In light of marine bacterial biodiversity and the urgent requirement for antibiotic alternatives, we can assume that the marine bacteriocin category will grow exponentially in the near future.

### 3.2. Bacteriocin classification

To date, about two hundred bacteriocins have been characterized (BACTIBASE, BAGEL). Bacteriocin classification is not well-established yet and is still the subject of debate. Although dating back to 1993, the bacteriocin classification defined by Klaenhammer is still the most cited one [62]. An update was proposed by Cotter *et al.* in 2005 [63] and debated by Heng and Tagg in 2006 [64,65]. Bacteriocins are usually classified combining various criteria. The main ones being the producer bacterial family, their molecular weight and finally their amino acid sequence homologies and/or gene cluster organization. An overview of bacteriocins known to date, proposed in Table 1, shows two main categories: the protein-bacteriocins mainly produced by *Gracilicutes*, mostly enterobacteriae and the peptide-bacteriocins from *Firmicutes*, chiefly from LAB. Even so, this statement needs to be qualified since enterobacteriae and LAB were the main bacteria studied for bacteriocin production. Our feeling is that peptide bacteriocins from *Gracilicutes* such as microcins are no exceptions.

Colicins are protein-bacteriocins containing about 500–600 amino acid residues [66]. They are organized in three specific domains. Binding to a specific receptor of the target cell, which is the first step of colicin cytotoxic action is governed by the central domain of colicins. The *N*-terminal and *C*-terminal domains are respectively responsible for colicin translocation and antibacterial activity (for a review see [67]). They have been classified in two sub-classes, based on cross resistance [68], translocation system, mechanism of release from the producing cell, and size of encoding plasmids [69]. Group A, translocated by the Tol system and encoded by small plasmids, is composed of colicins A, E1 to E9, K, L, N, S4, U, and Y while group B, translocated by the TonB system and encoded by large plasmids, are made up of colicins B, D, H, Ia, Ib, M, 5, and 10.

Both groups act on sensitive cells by targeting either the inner membrane by pore formation or an intracellular target using enzymatic activity such as DNAse or RNAse [67]. Bacteriocins of such molecular weight are exceptions in *Firmicutes* compared with the colicin family. Only two have been described in LAB [79,80]. Such protein-bacteriocins produced by LAB have been named class III bacteriocins. The others are specific of *Bacillus megaterium* [93], *Enterococcus faecalis* [81] or *Staphylococcus aureus* [82].

**Figure 2:**
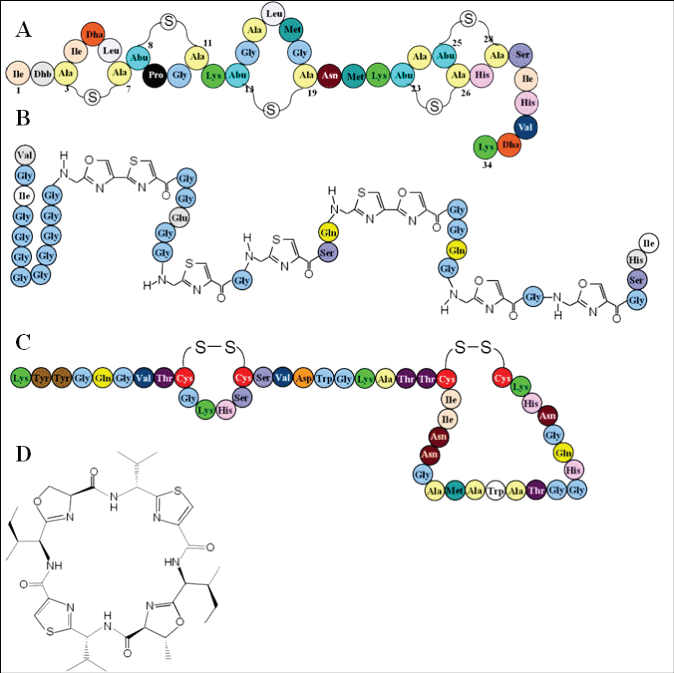
Covalent structure of some representative peptide-bacteriocins. A: nisin, B: microcin B17, C: pediocin PA-1, D patellamide A.

The peptide-bacteriocin group is produced by *Gracilicutes* and *Firmicutes* as well. Until 2007, the microcin group was composed of two classes, based on their post-translational modifications [94]. According to their gene cluster organization, this classification has recently evolved [35,59] to give birth to two main classes and two sub-classes. Class I comprises the smallest microcins with molecular masses ranging from 1.1 kDa to 3 kDa (Table 1). They display drastic post-translational modifications leading to exotic structures such as thiazole and oxazole rings in MccB17 (Figure 2). This class acts on sensitive cells by interaction with an intracellular target such as DNA gyrase inhibited by MccB17 [95]. The second microcin class is divided into two sub-classes. The microcin class IIa bridges the gap between colicin and microcin since these peptides are bigger (about 8 kDa) than a typical microcin and exhibit no modifications with the exception of a single disulfide bond formation. One of them, Microcin V (MccV) was previously called colicin V [35], the first documented bacteriocin [33]. Nevertheless, its gene cluster organization connects them undoubtedly to the microcin family [35]. Unlike previous microcins, class IIb microcins are chromosomally encoded, lacking disulfide bond, exposing a conserved serine-rich C-terminal and carrying for some of them a siderophore-type part (MccE492). MccE492 carries out its antibacterial activity by membrane permeabilization. But it was shown to target inner membrane proteins belonging to the mannose permease family [96].

The other main peptide bacteriocins family is the LAB one. Indeed, of the two hundred or so bacteriocins described to date, almost 90% are from LAB. With the exception of Helveticin J [79] and Milletricin [80], which are members of class III bacteriocins, they all are of peptidic nature. They have been divided into two main classes: class I and class II, the latter in turn containing three sub-classes (Table 1).

Lantibiotics have been defined as class I. Lantibiotic peptides undergo drastic posttranslational modification leading to unusual amino acid residues such as lanthionine. In a way, they are the counterpart of microcins in *Firmicutes*. To date, about 50 different lantibiotics have been described in LAB and non LAB bacteria such as *Staphylococcus aureus* [97]. Overall, lantibiotics are divided on the basis of their topology, that is to say their lanthionine bridge arrangements. Type-A lantibiotics such as nisin (Figure 2) are linear and cationic peptides, while type-B ones are globular [86,98]. The former exerts its antibacterial activity by membrane permeabilization by pore formation in a torroid manner [98] after binding to lipid II, while the latter targets intracellular enzyme function [98]. Another emerging lantibiotic class is the two-component lantibiotics such as haloduracin [99–101].

Class II bacteriocins are lightly modified peptides. These peptides are 20 to 70 amino acid residue-long. Extensive studies have been carried out about their mechanism of action. It has appeared that they use a common global procedure targeting a membrane-embedded domain of an integrated membrane protein [91]. The conformational modifications resulting from membrane protein–bacteriocin interactions lead to membrane perturbations, permeabilization and finally bacterial cell death [102]. It was divided into four sub classes on the basis of their activity. Class IIa was also named pediocin-like or anti-*Listeria* bacteriocins since all of them displayed antibacterial activity against *Listeria* spp. [62]. These bacteriocins are peptides sharing a highly conserved *N*-terminal part harboring a consensus sequence: -Y-Y-G-N-G-V-X-C-x-x-x-x-C (Figure 2) where C residues are involved in a disulfide bridge [48]. Their more variable C-terminal part has been used for their segregation in four sub-groups [63,102]. They act on target cells by a pore-forming mechanism of action [48,87,102]. This class constitutes the bacteriocin success story of the last twenty years. Class IIb is an original antimicrobial peptide class because it is made up of two independent peptides, each being active but both being required for optimal activity [102]. Around twelve such two-component bacteriocins have been described in LAB. Each time, the most active mix was obtained with equivalent concentration of each peptide [88]. LAB bacteriocin group IIc are real cyclic peptides since their *N*- and *C*-termini are covalently connected (for review, the reader is referred to [63,89]). Their mechanism of action when explored was permeabilization of the inner membrane of target cells leading to cell death. Finally, unmodified and non-pediocin-like peptides and single peptide active bacteriocins form class IId. To date, about 32 different class IId peptides have been described [102].

### 3.3. Bacteriocin specificity

Bacteriocins are unique antimicrobial peptides. Indeed, the producing strain has to protect itself from its own peptides, so bacteriocin-producing bacteria have to develop some sort of immunity strategy. In addition to a structural gene, post-translational gene and export machinery, the gene cluster organization of bacteriocin encodes as well for an immunity protein. The latter ensures bacteriocin protection in various ways, depending on the bacteriocin mechanism of action.

Immunity to pore forming colicins is mediated by a 11 to 18 kDa small membrane protein. A direct and specific interaction within the inner membrane between the immunity protein and the *C*-terminal part of colicin achieves cell protection. Transmembrane helices have been shown to be the main motifs recognized by immunity proteins. Colicins targeting intracellular enzymes such as nuclease are inactivated by direct binding of the immunity protein (about 10 kDa) to the active domain of colicin leading to a 71-kDa heterodimer.

Microcin immunity still remains opaque, while that towards lantibiotic has been recently reviewed [103,104]. Lantibiotic immunity is conferred by lipoprotein intercepting lantibiotic at the cytoplasmic membrane and/or ABC transporter–type membrane protein complex. Immunity to class II bacteriocins produced by LAB has recently been cleared up [91]. It implies that components of the mannose phosphotransferase system are receptors for both bacteriocin and the imunity protein [105]. To define the role of bacteriocins in producing bacteria is still a challenge. Its production entails advantages in colonizing or defending ecological niches for producing bacteria.

## 4. Marine Animal-Associated Microorganisms as Bacteriocin Producers

Marine animal-associated micro-organisms have been recently studied. Various authors have shown that these bacteria belong to the genera *Vibrio*, *Pseudoalteromonas*, *Aeromonas*, *Alteromonas*, and to the *Cytophaga*- *Flavobacterium*- *Bacteroides* group [106,107]. Currently, there are relatively few reports in the literature of antibacterial peptide or proteins produced by marine bacteria that have identified step sequence/structure. Wilson *et al.* [107] have isolated eight marine bacteria which produced antibacterial substances from a variety of different marine invertebrates (oysters, barnacles, sponges, tunicates, sea urchins, seaweeds). The loss of activity, after proteolytic digestion of their extracts, has suggested a proteinceous nature.

An increasing number of compounds with antibacterial activity have been found to be produced by a variety of organisms present in the marine surface environment. Potentially, there are many cases in which products previously attributed to higher organisms may be produced by their associated microorganisms such as patellamide [92]. Finally, numerous studies have evaluated antimicrobial marine isolates from sponge, coral, alga and mollusc associated bacteria [106–108]. Nevertheless, only a few studies have focused on marine bacterium isolation from marine animals and the search for their ability to produce bacteriocins (Table 2).

### 4.1. BLIS from *Vibrio* sp

*Vibrio* species are ubiquitous in the marine environment and are commonly isolated from fish and shellfish specimens [109]. Some species may be pathogenic to marine life, but some do not appear to affect them. Due to their capability to occupy this ecological niche they have been studied for their capacity to produce bacteriocin-like inhibitory substances (BLIS). Zai *et al.* [110] have isolated and identified fifty strains of the genus *Vibrio* isolated from the gills and gut region of healthy and infected catfishes (*Arianus thalassinus*). BLIS was detected and called Vibriocin AVP10 (Table 2).

Fresh and frozen seafood were studied by Carraturo *et al*. [111]. They have isolated three non-pathogenic (for humans) species of *Vibrio* (*V. mediterranei* 1, *V. mediterranei* 4 and *V. fluvialis*) displaying antagonistic activity on solid agar medium against pathogenic *V. parahaemolyticus* and *V. mediterranei*. A partial purification of a BLIS produced by *V. mediterranei* 1 was reported. Its proteinaceous nature was revealed by enzymatic degradation by proteinase K. Thanks to size exclusion chromatography, Carraturo *et al*. [111] have purified an antimicrobial fraction whose molecular mass was determined by SDS-PAGE to be 63–65 kDa corresponding to a mixture of unrelated polypeptides, including the bacteriocin.

Furthermore, *V. harveyi* is a serious pathogen of many vertebrate and invertebrate marine animals [112,113]. McCall and Sizemore [114] have reported for the first time the production of a bacteriocin in a strain of *Beneckea harveyi* (*V. harveyi*). The bacteriocin, ‘harveyicin SY’, with an estimated molecular mass of 24 kDa, was lethal to two strains of *V. harveyi*, KN96 and BBP8 (Table 2). Harveyicin SY was susceptible to proteolytic enzymes, and is apparently plasmid associated [114,115].

Prasad *et al*. [112], whilst screening various *V. harveyi* isolates from their culture collection have recognized a possible BLIS production by a strain of *V. harveyi* (VIB 571). Interestingly, this strain has been demonstrated to be pathogenic to rainbow trout (*Oncorhynchus mykiss*) and Atlantic salmon (*Salmo salar*) [113].

Inter-strain and inter-species inhibition mediated by a bacteriocin-like inhibitory substance (BLIS) from *V. harveyi* VIB 571 was demonstrated against four isolates of the same species and *V. fischeri*, *V. gazogenes* and *V. parahaemolyticus* (Table 2). The crude BLIS, which was obtained by ammonium-sulphate precipitation of the cell-free supernatant of a 72 h broth culture, was inactivated by lipase, proteinase K, pepsin, trypsin, pronase E and SDS. Incubation for 10 min at more than 60 °C resulted in loss of activity. On the other hand, antibacterial activity was not affected by pH. Anion-exchange chromatography, gel filtration, SDS-PAGE and two-dimensional gel electrophoresis revealed the presence of a single major peak, comprising a protein with a pI of ~5.4 and a molecular mass of ~32 kDa (Table 2). The N-terminal sequencing of the ~32 kDa protein yielded: D-E-Y-I-S-X-N-K-XS-S-A-D-I where ‘X’ may be cystein or modified amino acid residues.

Other vibriocins were isolated by Shehane and Sizemore [116]. Their aim was to identify bacteriocins effective against *V. vulnificus* in seafood. Isolates from estuaries near Wilmington (NC, USA) containing plasmids were checked for antimicrobial activity which was not due to lytic bacteriophage or small, non specific molecules. Three bacteriocin producers of *V. vulnificus* were detected and their inhibitory spectra determined (Table 2). Strain IW1 inhibited few strains of *V. vulnificus*; BC1 inhibited several strains of *V. vulnificus*, *V. cholerae* and *V. parahaemolyticus* and BC2 inhibited all tested *Vibrio* spp, *Plesiomonas shigelloides* and *E. coli*. Loss of inhibitory activity coincided with loss of the bacteriocinogenic plasmid. The molecular weights of the bacteriocins were estimated to be 9.0 kDa for IW1, 7.5 kDa for BC1 and 1.35 kDa for BC2 thanks to size exclusion chromatography. IW1 was heat labile, while BC1 was moderately stable except at extreme temperatures. BC2 was very stable and maintained its activity when frozen, autoclaved or exposed to extreme pH values [116]. The authors suggested that these bacteriocins might provide a tool for the removal of *V. vulnificus* from seafood.

Strain *Vibrio* sp. NM 10 was isolated from spotnape ponyfish (*Leiognathus nuchalis*) collected in coastal regions of Enoshima Island, Kanagawa, Japan. This strain exhibited high activity against *P. piscicida* K-III, but was also able to inhibit *E. coli* IAM 1264, *V. vulnificus* RIMD 2219009 and *Enterococcus seriolicida* YT-3 [117]. The antibacterial substance produced by *Vibrio* sp. NM 10 is a proteinaceous heat-labile substance with a molecular mass of less than 5 kDa. These facts strongly suggest that the antibacterial substance is either a bacteriocin or a bacteriocin-like substance [117].

### 4.2. BLIS from marine *Aeromonas* sp

Authors Moro *et al*. [118] and Messi *et al*. [119] have shown their interest in evaluating BLIS production in *Aeromonas hydrophila*. All strains of *Aeromonas hydrophila* in these two studies demonstrated inhibitory activities against several strains of *Staphylococcus aureus* (Table 2). Messi *et al*. [119] have demonstrated further inhibitory effect against *Listeria species, Streptococcus agalactiae* and *Lactobacillus* sp. No inhibition was observed against all Gram-negative strains assayed, including related species (*Aeromonas sobria* ATCC 43979, *A. caviae* ATCC 13137). Such an inhibitory spectrum is not compatible with the bacteriocin definition.

### 4.3. BLIS from marine *Pseudoalteromonas* sp

Longeon *et al*. [121] investigated bacteria collected from different substrates on the littoral of Brittany and they focused their attention on a *Pseudoalteromonas* sp. named X-153 that exhibited high antimicrobial activity. Purification of the active protein P-153 from the bacterial cells was achieved. This antibacterial protein was evaluated by size exclusion chromatography to be of 280 kDa size. This antibacterial protein was shown to be active against both gracilicutes (ichthyopathogenic *Vibrio*) and firmicutes (*Staphylococcus epidermidis, Propionibacterium acnes* and *P. granulosum*) (Table 2). Such a broad spectrum of activity is not consistent with the definition of a bacteriocin.

### 4.4. Bacteriocin from Firmicutes and LAB associated to marine animals

It is generally considered that Gram-positive bacteria, including lactic acid bacteria, are numerically dominant members of the normal microbiota in the gastrointestinal tract of endothermic animals at an early stage of their lives [122]. The gastrointestinal microbiota of healthy fish is usually composed of lactic acid bacteria belonging to the genera *Streptococcus, Lactobacillus, Carnobacterium, Leuconostoc* [122]. Divercins and piscicocins have been fully characterized from *Carnobacterium* isolated from fish intestine (Table 3). These two bacteriocins belong to class IIa of bacteriocins produced by LAB (see Table 1, for review the reader is referred to [123]).

In 2004, Pirzada *et al*. [120] isolated and studied a bacteriocinogenic strain ZM81, a Gram positive pleomorphic rod, which was isolated from the open sea region of Karachi. The proteinaceous nature of the cell-free supernatant of marine strain ZM81 was defined by enzyme degradation with pronase and trypsin. Fractionization of the crude bacteriocin thanks to a molecular weight cut-off membrane showed an enrichment of activity in the fraction containing >10 kDa bacteriocin-like inhibitory substance. BLIS produced by Marine Bacterium ZM81 is heat labile and exhibits activity within a wide pH range of 4–12 [120].

### 4.5. Bacteriocin from marine cyanobacteria

While most small peptides found in *Cyanobacteria* are biosynthesized by nonribosomal peptide synthetases [131], a microcin-like pathway for the biosynthesis of a family of cyclic peptides, the patellamides (Figure 2), has been recently reported in *Prochloron didemni*, a cyanobacterial symbiont of tropical ascidians [92]. The patellamides are moderately cytotoxic and composed of a pseudosymmetrical, cyclic dimer, with each substructure having the sequence thiazole-nonpolar amino acid-oxazoline-nonpolar amino acid. Despite these unusual features, patellamide biosynthesis is ribosomal [132]. The discovery of patellamides has provided first insight into the biosynthesis of microcin-like peptide distribution and versatility in *Cyanobacteria* [133].

The patellamide family are cyclic octapeptides (Figure 2) characterized by the presence of thiazole and oxazole moieties. Although nonribosomal biosynthesis was anticipated for the formation of these peptides, heterologous expression of a microcin-like gene cluster discovered in the genome of the cyanobacterium *Prochloron didemni* unambiguously showed that these peptides are produced by a ribosomal pathway [92,133,134]. An increasing number of other cyclic peptides containing heterocyclic amino acids has recently been isolated from planktonic and other animal-associated *cyanobacteria*, including nostocyclamide [135], tenuecyclamides [136], venturamides [137], dendroamides [138], and microcyclamides [139]. The variety of structures is reflected in an equally large variety of bioactivities, such as antibacterial, cytotoxic, and antimalarial activities [133].

## 5. Bacteriocin-Based Strategy to Select a Probiotic for Aquaculture

In animal or human nutrition, lactic acid bacteria largely dominate the worldwide probiotic market. Actually, they have demonstrated their safety and efficiency over a century. Aquaculture is no exception. Indeed most probiotics used in aquaculture belong to the LAB (for a review the reader is referred to [140,141]). *Bacillus* genus has also been successfully used in aquaculture [142] makes the list longer by adding marine bacteria belonging to *Aeromonas, Pseudomonas, Pseudoalteromonas, Roseobacter* and *Vibrio* and a yeast, *Saccharomyces cerevisiae* [143]. Moreover, the potential of marine actinobacteria as probiotics in aquaculture has been recently reviewed [144]. All the same, marine bacteria are much less developed as a probiotic source in spite of promising results [143,144]. This may be due to a dissuasive legislation.

The use of probiotics in aquaculture is closely controlled by the administration. In the European Union, probiotics are controlled by regulation CE n°1831/2003 (OJ L 268 of 18.10.2003) on the use of additives in animal food. Industrialists have to obtain an authorization from the European Food Safety Authority (EFSA), which controls the beneficial effects of probiotics on animal and human health safety and the environment. The safety assessment of microorganisms is in a guideline issued by a relevant scientific committee (SCAN) in 2001. Several specific tests and studies have to be carried out in order to obtain the QSP (Qualified Presumption of Safety) [145]. In the US, probiotics which are used in animal feed are called “direct fed microbials” and are regulated by the Food and Drug Administration. The efficacy and safety of probiotics are examined by the FDA, which can recognize the microorganism as safe (Generally Recognised As Safe). The GRAS-status can be obtained in two ways: some microorganisms have a long history of safety (find them at http://www.cfsan.fda.gov/~dms/opa-micro.html.) or have been recognized by qualified experts as safe under the conditions of intended use [145]. The GRAS concept means that responsibility for safety of the products resides strictly with the producer. To obtain probiotic authorization, the manufacturer has to send a petition to the FDA and the general requirements for this petition are detailed in the US Code of Federal Regulations [146].

**Figure 3:**
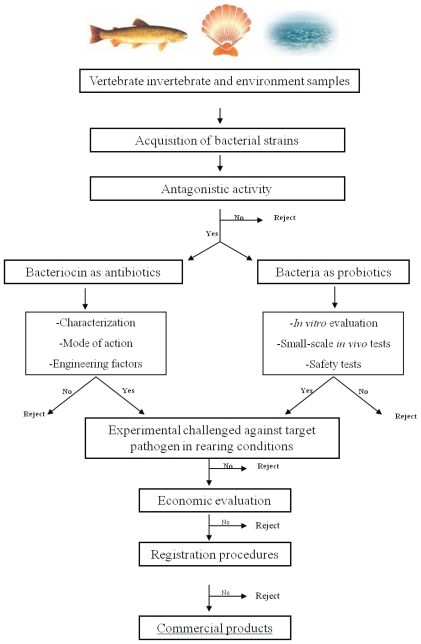
Strategy to select probiotics for aquaculture.

We advocate a strategy aiming at preventing establishment of pathogenic bacteria using probiotics. These should be selected from natural indigenous microbial communities associated with marine animals. Bacteriocins are efficient weapons to protect and thus to defend an ecological niche or a nutrient pool. Indeed these peptides combine the most potent activity of related bacteria and a specific spectrum of activity [45]. Thus they constitute a pertinent tool to select a probiotic. So we propose a strategy for probiotic selection based on bacteriocin production ability (Figure 3).

The first step of selection is to screen the animal-associated micro-organisms for antagonistic activities against the selected target cells. This acquisition is one of the major phases of assessment of potential probiotics [147]. Probiotic bacteria for use in aquaculture have to be isolated from indigenous or exogenous microbiota of aquatic vertebrates or invertebrates [27] in order to facilitate establishment and efficiency over temperature and salinity variations of aquaculture farming [144]. This selection is generally carried out by using *in vitro* antagonist tests [25,144]. Then two ways are possible: the use of the inhibitory compounds as an antibiotic or that of bacteria as probiotics (Figure 3). For the former, research has to be carried out in order to determine the nature of inhibitory compounds, the mode of action, and the genetic aspect of BLIS. However, the administration of purified bacteriocins does not appear to be a cost-effective approach. In face of this need, the second alternative seems to be a more feasible approach. When the putative probiotic is selected, the research has to focus on two parts: the evaluation of safety and the beneficial effects under rearing conditions [147]. Safety has to be proved under *in-vivo* and rearing conditions for the host of course, but also for the environment. Then the commercial procedures can be submitted to the authorities concerned.

It is important to note that probiotics producing antagonistic compounds have to be used in a preventive way [148]. Indeed, a review [149] showed that BS107 (a marine bacteria identified as *Roseobacter*) cannot be used for treatment when the pathogen is in sufficient concentration to provoke a disease outbreak. In fact, the authors demonstrate that BS107 (10^6^ cells/mL) doesn’t have any probiotic activity when living cells are co -inoculated with *Vibrio pectenicida* A496 (10^4^ cells/mL).

## 6. Conclusions

Bacteriocins from LAB have demonstrated their remarkable potential as food conservatives [13,44–50,63,87,123], or as therapeutics for veterinary or medical uses [13,53] or as phytosanitary for plant protection [51]. Extensively studied in LAB and enterobacteriae, little or nothing is known of marine bacteriocins. Only a few have been described, and the fully-characterized ones are exceptions, so knowledge of marine bacteriocins is at the early stage s.

Moreover, the increasing scarcity of marine resources ensures a rise in aquaculture in the next decades but also condemns it to set up effective strategies respectful of the environment. In this context, marine bacteriocins that are produced by LAB or autochthonous associated marine bacteria seem to be a relevant alternative to antibiotics. Based on both bacteriocin diversity described to date and the ocean microbial biodiversity, one can predict the discovery of a true peptide arsenal in the coming years. Such an arsenal will find applications in aquaculture. Indeed, the antibiotic approach has to be redrawn. The approach that we recommend rests on different concepts: (i) the most effective strategy to limit epizooties consists in avoiding contact between host and pathogen; (ii) to eradicate pathogenic bacteria is illusory, it’s better to occupy its ecological niche (iii) in the case of infections, it is preferable to use active ingredients with a narrow spectrum of antibacterial activity so as to better target the pathogenic ones and limit the risk of resistance development. Bacteriocins are perfect tools to select as probiotics to apply this strategy. One can dream of protective probiotics made up of several BLIS-producing bacteria acting synergistically against pathogens.

## Figures and Tables

**Table 1:** Bacteriocin overview.

(A)						
Protein-Bacteriocins	Class	Sub-Class	Name	MM (kDa)	Mode of action	Ref.
**Gracilicutes**						
*Escherichia coli*	Colicins	Groupe A		40 to 80	Nuclease/Pore-forming	[69]
		Groupe B		40 to 80	Nuclease/Pore-forming	[69]
*Pseudomonas aeruginosa*	Pyocins	R-type	Pyocin R2	270 (AA)	Pore-forming	
		S-type	Pyocin S1,S2,AP41	75/84/94	Phage-tail like	[70]
		F-type	Pyocin F		Phage-tail like	
*Hafnia alvei*	Alveicins	Colicin like	Alveicin A, B	408/358 (AA)	Pore forming	[71]
*Klebsiella pneumonia*	Klebicin	Colicin-like	Klebicin C, D	96	Nuclease	[72,73]
*Serratia plymithicum*	Serracin		Serracin P	66	Phage-tail like	[74]
*Xanthomonas campestris*	Glynericin		Glynericin A	50	Phage tail like	[75,76]
*Yersinia enterocolitica*	Enterocoliticin			669	Phage tail like	[77]
*Erwinia carotovora*	Carotovoricin		Carotovoricin Er	68/76	Phage tail like	[78]
**Firmicutes**						
*Lactobacillus helveticus*	Helveticin J	Class III		37,5	to be defined	[79]
*Streptococcus milleri*	Millericin	Class III		30	Peptidoglycan hydrolysis	[80]
*Enterococcus faecalis*	Enterolysin	Class III		34,5	Peptidoglycan hydrolysis	[81]
*Staphylococcus aureus*	Lysostaphin	Class III		25	Peptidoglycan hydrolysis	[82,83]

(B)								
Peptide-Bacteriocin	Class	Sub-Class		Name	MM (kDa)	PTM	Mode of action	Ref.
**Gracilicutes**								
*Escherichia coli*	Microcin	Class I		Microcin B17	3.1	drastic	intracellular enzymes	
		Class II	IIa	Microcin V	8.8	light	pore-forming	[35,59,61]
			IIb	Microcin E492	7.9	drastic	pore forming	
**Firmicutes**								
Lactic acid bacteria	Class I	A-type	A1	Nisin	3.5	drastic	pore-forming	[84,85]
(mainly)	or Lantibiotic		A2	Lacticin 481	3	drastic	pore forming	[86]
		B-type		Mersacidin	2			[61]
	Class II	class IIa		Pediocin	4.6	light	pore forming	[48,87]
		class IIb		Plantaricin E/F	3.5/3.7	light	pore forming	[88]
		Class IIc		carnocyclin A	5.8	cyclic	pore forming	[89,90]
		Class IId		Lactococcin A	5.8	none	pore forming	[91]
***Cyanobacteria***								
*Prochloron didemni*	microcin –like	-		Patellamides	0.7	drastic		[92]

Ref., PTM, AA and ref. respectively mean Review reference, Post-translational modification and amino acids.

**Table 2:** Bacteriocins produced by bacteria isolated from marine environment.

Producing strain	Bacteriocin	Inhibited strain(s)	Isolated from	MM (kDa)	Ref.
*Listonella anguillarum* AVP10	Vibriocin AVP10	*Escherichia coli* *Listonella anguillarum* AVS9^1^	Healthy and infected catfishes (*Arius thalassimus*)	?	[110]
*Vibrio mediterranei*	BLIS	*V. parahaemolyticus* *V. mediterranei* 5	Fresh & frozen seafood	63–65^a^	[111]
*Vibrio harveyi* VIB 571	BLIS	*Vibrio harveyi*^1^ *V. fischeri* *V. gazogenes* *V. parahaemolyticus*	-	~32^a^,^b^	[112]
*Vibrio harveyi (Beneckea harveyi* SY)	Harveyicin SY	*V. harveyi*^1^	area of Galveston Island	24	[114,115]
*Vibrio vulnificus*	IW1	*V. vulnificus* *V. cholera*	Water samples from Wilmington (NC, USA)	9	[116]
	BC1	*V. parahaemolyticus*		7,5	
	BC2	*Vibrio* spp. *Plesiomonas shigelloides* *E. coli*		1,35	
*Vibrio* sp. Strain NM 10	BLIS	*Pasteurella piscicida* K-III; *E. coli*; *V. vulnificus* *Enterococcus seriolicida*	*Leiognathus nuchalis* intestine	< 5^d^	[117]
Bacteriocinogenic strain marine strain ZM81 (Gram positif pleomorphic strain)	Bacteriocins/BLIS	Marine bacterial strain ZM19	Open sea region of Karachi coast	>10	[120]
*Aeromonas hydrophila*	BLIS	*Staphylococcus aureus* strains	Water tank containing alligators	?	[118] [119]
*Pseudoalteromonas* Species Strain X153	Antibiotic protein P-153	Ichthyopathogenic *Vibrio*^1^ *Staphylococcus epidermidis* *Propionibacterium acnes* *Propionibacterium granulosum*	Substrates on the littoral of Brittany	280^a^,^b^	[121]

a Molecular mass was evaluated using sodium dodecyl sulfate polyacrylamide gel electrophoresis;

b size-exclusion chromatography,

c Mass Spectrometry or

d ultrafiltration.

1: aquacole pathogen.

2: bacteriocin isolated from fish intestine.

?: Unknown molecular mass.

**Table 3:** Bacteriocin produced by Lactic Acid Bacteria isolated from marine animal.

Producing strain	Bacteriocin	Inhibited strain(s)	Isolated from	MM (kDa)	Ref.
*Enterococcus faecium* LHICA 28.4, 34.5, 40.4, 46	Enterocin P	*Carnobacterium maltaromaticum* *Listeria monocytogenes* *Staphylococcus aureus*	Turbot muscle		[124]
*Enterococcus faecium* ALP7	bac ALP7	*Listeria monocytogenes*	Non-fermented shellfish including oysters, mussels and clams	<10	[125]
*Pediococcus pentosaceus* ALP57	bac ALP57	*Bacillus subtilis* *Enterococcus faecalis* *Lactobacillus brevis gravensis;* *Lactobacillus curvatus* *Listeria innocua*			
*Carnobacterium divergens* V41	Divercin V41	*Listeria monocytogenes*	Salmon intestine	4,509	[126–129]
*Carnobacterium piscicola* V1	Piscicocin V1a Piscicocin V1b	*Listeria monocytogenes*	Trout intestine	4,416 4,526	[128,130]

## Abbreviations

APD2: Antimicrobial peptide database 2
BLIS: Bacteriocin-like inhibitory substance
FDA: Food and Drug Administration
GRAS: Generally recognize as safe
LAB: Lactic acid bacteria

## References

[1] Kurath G Biotechnology and DNA vaccines for aquatic animals Rev Sci Tech Off Int Epiz 2008 27 175 196 18666487

[2] Toranzo AE Magariños B Romalde JL A review of the main bacterial fish diseases in mariculture systems Aquaculture 2005 246 37 61

[3] Austin B Zhang XH Vibrio harveyi: a significant pathogen of marine vertebrates and invertebrates Lett Appl Microbiol 2006 43 119 124 16869892 10.1111/j.1472-765X.2006.01989.x

[4] Paillard C Le Roux F Borrego JJ Bacterial disease in marine bivalves, a review of recent studies: Trends and evolution Aquat Living Res 2004 17 477 498

[5] Marcogliese D The impact of climate change on the parasites and infectious diseases of aquatic animals Rev Sci Tech Off Int Epiz 2008 27 467 484 18819673

[6] Cabello FC Heavy use of prophylactic antibiotics in aquaculture: a growing problem for human and animal health and for the environment Environ Microbiol 2006 8 1137 1144 16817922 10.1111/j.1462-2920.2006.01054.x

[7] Zhou Q Li K Jun X Bo L Role and functions of beneficial microorganisms in sustainable aquaculture Bioresour Technol 2009 100 3780 3786 19261470 10.1016/j.biortech.2008.12.037

[8] Dorrington T Gomez-Chiarri M Antimicrobial Peptides for Use in Oyster Aquaculture: Effect on Pathogens, Commensals, and Eukaryotic Expression Systems J Shellfish Res 2008 27 365 374

[9] Kesarcodi-Watson A Kaspar H Lategan MJ Gibson L Probiotics in aquaculture: The need, principles and mechanisms of action and screening processes Aquaculture 2008 274 1 14

[10] Joerger RD Alternatives to antibiotics: bacteriocins, antimicrobial peptides and bacteriophages Poult Sci 2003 82 640 647 12710486 10.1093/ps/82.4.640

[11] Gillor O Etzion A Riley MA The dual role of bacteriocins as anti- and probiotics Appl Microbiol Biotechnol 2008 81 591 606 18853155 10.1007/s00253-008-1726-5 PMC2670069

[12] Gillor O Nigro LM Riley LM Genetically Engineered Bacteriocins and their Potential as the Next Generation of Antimicrobials Curr Pharm Des 2005 11 1067 1075 15777256 10.2174/1381612053381666

[13] Lenski RE Riley MA Chemical warfare from an ecological perspective Proc Natl Acad Sci USA 2002 99 556 558 11805313 10.1073/pnas.022641999 PMC117343

[14] Riley MA Gordon DM The ecological role of bacteriocins in bacterial competition Trends Microbiol 1999 7 129 133 10203843 10.1016/s0966-842x(99)01459-6

[15] Riley MA Wertz JE BACTERIOCINS: Evolution, Ecology, and Application Annu Rev Microbiol 2002 56 117 137 12142491 10.1146/annurev.micro.56.012302.161024

[16] Kollath W Nutrition and the tooth system; general review with special reference to vitamins Dtsch Zahnarztl Z 1953 8 Suppl 7 16 13068115

[17] Parker RB Probiotics, the other half of the antibiotic story Anim Nutr Health 1974 29 4 8

[18] Fuller R Probiotics in man and animals J Appl Microbiol 1989 66 365 378 2666378

[19] Salminen S Ouwehand A Benno Y Lee YK Probiotics: how should they be defined Trends Food Sci Technol 1999 10 107 110

[20] Ouwehand AC Salminen SJ The health effects of cultured milk products with viable and non-viable bacteria Int Dairy J 1998 8 749 758

[21] Reid G Sanders ME Gaskins HR Gibson GR Mercenier A Rastall R Roberfroid M Rowland I Cherbut C Klaenhammer TR New Scientific Paradigms for Probiotics and Prebiotics J Clin Gastroenterol 2003 37 105 118 12869879 10.1097/00004836-200308000-00004

[22] Moriarty DJW Control of luminous Vibrio species in penaeid aquaculture ponds Aquaculture 1998 164 351 358

[23] Cahill MM Bacterial flora of fishes: A review Microb Ecol 1990 19 21 41 10.1007/BF02015051 24196252

[24] Jorquera MA Silva FR Riquelme CE Bacteria in the culture of the scallop Argopecten purpuratus (Lamarck, 1819) Aquaculture Int 2001 9 285 303

[25] Verschuere L Rombaut G Sorgeloos P Verstraete W Probiotic Bacteria as Biological Control Agents in Aquaculture Microbiol Mol Biol Rev 2000 64 655 671 11104813 10.1128/mmbr.64.4.655-671.2000 PMC99008

[26] Tinh N Dierckens K Sorgeloos P Bossier P A review of the functionality of probiotics in the larviculture food chain Mar Biotechnol 2008 10 1 12 18040740 10.1007/s10126-007-9054-9

[27] Balcázar JL Blas Id Ruiz-Zarzuela I Cunningham D Vendrell D Múzquiz JL The role of probiotics in aquaculture Vet Microbiol 2006 114 173 186 16490324 10.1016/j.vetmic.2006.01.009

[28] Bomba A Nemcová R Mudronová D Guba P The possibilities of potentiating the efficacy of probiotics Trends Food Sci Technol 2002 13 121 126

[29] Musa HH Wu SL Zhu CH Seri HI Zhu GQ The Potential Benefits of Probiotics in Animal Production and Health J Anim Vet Adv 2009 8 313 321

[30] Isolauri E Sutas Y Kankaanpaa P Arvilommi H Salminen S Probiotics: effects on immunity Am J Clin Nutr 2001 73 444S 450S 11157355 10.1093/ajcn/73.2.444s

[31] Kelly D Conway S Aminov R Commensal gut bacteria: mechanisms of immune modulation Trends Immunol 2005 26 326 333 15922949 10.1016/j.it.2005.04.008

[32] Cornil V Babes V Les bactéries et leur rôle dans l’anatomie et l’histologie pathologiques des maladies infectieuses: ouvrage contenant les méthodes spéciales de la bactériologie F. Alcan Paris, France 1885

[33] Gratia A Sur un remarquable exemple d’antagonisme entre deux souches de colibacille CR Soc Biol 1925 93 1040 1041

[34] Fredericq P Joiris E Betz-Barreau M Gratia A Recherche des germes producteurs de colicines dans les selles de malades atteints de fièvre paratyphoide C R Soc Biol 1949 143 556 559

[35] Duquesne S Destoumieux-Garzon D Peduzzi J Rebuffat S Microcins, gene-encoded antibacterial peptides from enterobacteria Nat Prod Rep 2007 24 708 734 17653356 10.1039/b516237h

[36] Jacob F Lwoff A Siminovitch A Wollman E Définition de quelques termes relatifs à la lysogenie Ann Inst Pasteur 1953 84 222 224 13031254

[37] Bradley DE Ultrastructure of phages and bacteriocins Bacteriol Rev 1967 31 230 314 4865539 10.1128/br.31.4.230-314.1967 PMC408286

[38] Reeves P The bacteriocins Bacteriol Rev 1965 29 24 45 14295984 10.1128/br.29.1.24-45.1965 PMC441259

[39] Rogers LA The inhibitory effect of Streptococcus lactis on Lactobacillus bulgaricus J Bacteriol 1928 16 321 325 16559344 10.1128/jb.16.5.321-325.1928 PMC375033

[40] Jansen EF Hirschmann DJ Subtilin, an antibacterial product of Bacillus subtilis: culturing conditions and properties Arch Biochem 1944 4 297 304

[41] Gross E Kiltz HH Nebelin E Subtilin, VI: the structure of subtilin (author’s transl) Hoppe Seylers Z Physiol Chem 1973 354 810 812 4154277

[42] Gross E Morell JL Nisin. The assignment of sulfide bridges of beta-methyllanthionine to a novel bicyclic structure of identical ring size J Am Chem Soc 1970 92 2919 2920 5439977 10.1021/ja00712a055

[43] Anonymous Nisin preparation: Affirmation of GRAS status as a direct human food ingredient Fed Regist 1988 Part 184 53 11247 11251

[44] Abee T Krockel L Hill C Bacteriocins: modes of action and potentials in food preservation and control of food poisoning Int J Food Microbiol 1995 28 169 185 8750665 10.1016/0168-1605(95)00055-0

[45] Deegan LH Cotter PD Hill C Ross PR Bacteriocins: Biological tools for bio-preservation and shelf-life extension Int Dairy J 2006 16 1058 1071

[46] Gálvez A Abriouel H López RL Omar NB Bacteriocin-based strategies for food biopreservation Int J Food Microbiol 2007 120 51 70 17614151 10.1016/j.ijfoodmicro.2007.06.001

[47] Nes IF Johnsborg O Exploration of antimicrobial potential in LAB by genomics Curr Opin Biotechnol 2004 15 100 104 15081046 10.1016/j.copbio.2004.02.001

[48] Papagianni M Anastasiadou S Pediocins: The bacteriocins of Pediococci. Sources, production, properties and applications Microb Cell Fact 2009 8 3 19133115 10.1186/1475-2859-8-3 PMC2634753

[49] Juncioni de Arauza L Jozalaa AF Mazzolab PG Vessoni Pennaa TC Nisin biotechnological production and application: a review Trends Food Sci Technol 2009 20 146 154

[50] Papagianni M Ribosomally synthesized peptides with antimicrobial properties: biosynthesis, structure, function and applications Biotechnol Adv 2003 21 465 499 14499150 10.1016/s0734-9750(03)00077-6

[51] Holtsmark I Eijsink VG Brurberg MB Bacteriocins from plant pathogenic bacteria FEMS Microbiol Lett 2008 280 1 7 18070073 10.1111/j.1574-6968.2007.01010.x

[52] Vidaver AK Bacteriocins: the lure and the reality Plant Dis 1983 67 471 474

[53] Tagg JR Dierksen KP Bacterial replacement therapy: adapting ‘germ warfare’ to infection prevention Trends Biotechnol 2003 21 217 223 12727383 10.1016/S0167-7799(03)00085-4

[54] Hammami R Zouhir A Ben Hamida J Fliss I BACTIBASE: a new web-accessible database for bacteriocin characterization BMC Microbiol 2007 7 89 17941971 10.1186/1471-2180-7-89 PMC2211298

[55] de Jong A van Hijum SA Bijlsma JJ Kok J Kuipers OP BAGEL: a web-based bacteriocin genome mining tool Nucleic Acids Res 2006 34 W273 279 16845009 10.1093/nar/gkl237 PMC1538908

[56] Wang G Li X Wang Z APD2: the updated antimicrobial peptide database and its application in peptide design Nucleic Acids Res 2009 37 D933 937 18957441 10.1093/nar/gkn823 PMC2686604

[57] Wang Z Wang G APD: the Antimicrobial Peptide Database Nucleic Acids Res 2004 32 D590 592 14681488 10.1093/nar/gkh025 PMC308759

[58] Wang CK Kaas Q Chiche L Craik DJ CyBase: a database of cyclic protein sequences and structures, with applications in protein discovery and engineering Nucleic Acids Res 2008 36 D206 210 17986451 10.1093/nar/gkm953 PMC2239000

[59] Duquesne S Petit V Peduzzi J Rebuffat S Structural and functional diversity of microcins, gene-encoded antibacterial peptides from enterobacteria J Mol Microbiol Biotechnol 2007 13 200 209 17827970 10.1159/000104748

[60] Severinov K Semenova E Kazakov A Kazakov T Gelfand MS Low-molecular-weight post-translationally modified microcins Mol Microbiol 2007 65 1380 1394 17711420 10.1111/j.1365-2958.2007.05874.x

[61] Jack RW Jung G Lantibiotics and microcins: polypeptides with unusual chemical diversity Curr Opin Chem Biol 2000 4 310 317 10826980 10.1016/s1367-5931(00)00094-6

[62] Klaenhammer TR Genetics of bacteriocins produced by lactic acid bacteria FEMS Microbiol Rev 1993 12 39 85 8398217 10.1111/j.1574-6976.1993.tb00012.x

[63] Cotter PD Hill C Ross RP Bacteriocins: developing innate immunity for food Nat Rev Microbiol 2005 3 777 788 16205711 10.1038/nrmicro1273

[64] Cotter PD Hill C Ross PR What’s in a name? Class distinction for bacteriocins Nat Rev Microbiol 2006 4 10.1038/nrmicro1273-c1

[65] Heng NCK Tagg JR What’s in a name? Class distinction for bacteriocins Nat Rev Microbiol 2006 4 10.1038/nrmicro1273-c1

[66] Riley MA Molecular mechanisms of bacteriocin evolution Annu Rev Genet 1998 32 255 278 9928481 10.1146/annurev.genet.32.1.255

[67] Cascales E Buchanan SK Duche D Kleanthous C Lloubes R Postle K Riley M Slatin S Cavard D Colicin biology Microbiol Mol Biol Rev 2007 71 158 229 17347522 10.1128/MMBR.00036-06 PMC1847374

[68] Davies JK Reeves P Genetics of resistance to colicins in *Escherichia coli* K12: cross-resistance among resistance of group A J Bacteriol 1975 123 102 117 1095546 10.1128/jb.123.1.102-117.1975 PMC235696

[69] Riley MA Wertz JE Bacteriocin diversity: ecological and evolutionary perspectives Biochimie 2002 84 357 364 12423779 10.1016/s0300-9084(02)01421-9

[70] Duport C Baysse C Michel-Briand Y Molecular characterization of pyocin S3, a novel S-type pyocin from Pseudomonas aeruginosa J Biol Chem 1995 270 8920 8927 7721800 10.1074/jbc.270.15.8920

[71] Wertz JE Riley MA Chimeric nature of two plasmids of Hafnia alvei encoding the bacteriocins alveicins A and B J Bacteriol 2004 186 1598 1605 14996789 10.1128/JB.186.6.1598-1605.2004 PMC355955

[72] James R Molecular Cloning and Purification of Klebicin B J Gen Microbiol 1988 134 2525 2533 2855528 10.1099/00221287-134-9-2525

[73] Riley MA Pinou T Wertz JE Tan Y Valletta CM Molecular characterization of the klebicin B plasmid of Klebsiella pneumoniae Plasmid 2001 45 209 221 11407916 10.1006/plas.2001.1519

[74] Jabrane A Sabri A Compere P Jacques P Vandenberghe I Van Beeumen J Thonart P Characterization of serracin P, a phage-tail-like bacteriocin, and its activity against Erwinia amylovora, the fire blight pathogen Appl Environ Microbiol 2002 68 5704 5710 12406768 10.1128/AEM.68.11.5704-5710.2002 PMC129874

[75] Heu S Oh J Kang Y Ryu S Cho SK Cho Y Cho M gly gene cloning and expression and purification of glycinecin A, a bacteriocin produced by Xanthomonas campestris pv. glycines 8ra Appl Environ Microbiol 2001 67 4105 4110 11526012 10.1128/AEM.67.9.4105-4110.2001 PMC93136

[76] Pham HT Riu KZ Jang KM Cho SK Cho M Bactericidal activity of glycinecin A, a bacteriocin derived from Xanthomonas campestris pv. glycines, on phytopathogenic Xanthomonas campestris pv. vesicatoria cells Appl Environ Microbiol 2004 70 4486 4490 15294776 10.1128/AEM.70.8.4486-4490.2004 PMC492317

[77] Strauch E Kaspar H Schaudinn C Dersch P Madela K Gewinner C Hertwig S Wecke J Appel B Characterization of enterocoliticin, a phage tail-like bacteriocin, and its effect on pathogenic Yersinia enterocolitica strains Appl Environ Microbiol 2001 67 5634 5642 11722917 10.1128/AEM.67.12.5634-5642.2001 PMC93354

[78] Nguyen HA Kaneko J Kamio Y Temperature-dependent production of carotovoricin Er and pectin lyase in phytopathogenic Erwinia carotovora subsp. carotovora Er Biosci Biotech Biochem 2002 66 444 447 10.1271/bbb.66.444 11999425

[79] Joerger MC Klaenhammer TR Cloning, expression, and nucleotide sequence of the Lactobacillus helveticus 481 gene encoding the bacteriocin helveticin J J Bacteriol 1990 172 6339 6347 2228964 10.1128/jb.172.11.6339-6347.1990 PMC526818

[80] Beukes M Bierbaum G Sahl HG Hastings JW Purification and partial characterization of a murein hydrolase, millericin B, produced by Streptococcus milleri NMSCC 061 Appl Environ Microbiol 2000 66 23 28 10618198 10.1128/aem.66.1.23-28.2000 PMC91780

[81] Nilsen T Nes IF Holo H Enterolysin A, a cell wall-degrading bacteriocin from enterococcus faecalis LMG 2333 Appl. Environ Microbiol 2003 69 2975 2984 12732574 10.1128/AEM.69.5.2975-2984.2003 PMC154489

[82] Kumar JK Lysostaphin: an antistaphylococcal agent Appl Environ Microbiol 2008 80 555 561 10.1007/s00253-008-1579-y 18607587

[83] Trayer HR Buckley CE Molecular properties of Lysostaphin, a bacteriolytic agent specific for staphylococcus aureus J Biol Chem 1970 245 4842 4846 5456157

[84] Brotz H Sahl HG New insights into the mechanism of action of lantibiotics--diverse biological effects by binding to the same molecular target J Antimicrob Chemother 2000 46 1 6 10882681 10.1093/jac/46.1.1

[85] Nagao J Asaduzzaman SM Aso Y Okuda K Nakayama J Sonomoto K Lantibiotics: insight and foresight for new paradigm J Biosci Bioeng 2006 102 139 149 17046525 10.1263/jbb.102.139

[86] Dufour A Hindré T Haras D Le Pennec J-P The biology of lantibiotics from the lacticin481 group is coming of age FEMS Microbiol Rev 2007 31 134 167 17096664 10.1111/j.1574-6976.2006.00045.x

[87] Drider D Fimland G Hechard Y McMullen LM Prevost H The continuing story of class IIa bacteriocins Microbiol Mol Biol Rev 2006 70 564 582 16760314 10.1128/MMBR.00016-05 PMC1489543

[88] Oppegård C Rogne P Emanuelsen L Kristiansen PE Fimland G Nissen-Meyer J The Two-Peptide Class II bacteriocins: structure, production, and mode of action J Mol Microbiol Biotechnol 2007 13 210 219 17827971 10.1159/000104750

[89] Maqueda M Sánchez-Hidalgo M Fernández M Montalbán-López M Valdivia E Martínez-Bueno M Genetic features of circular bacteriocins produced by Gram-positive bacteria FEMS Microbiol Rev 2008 32 2 22 18034824 10.1111/j.1574-6976.2007.00087.x

[90] Martin-Visscher LA Gong X Duszyk M Vederas JC The three-dimensional structure of carnocyclin A reveals that many circular bacteriocins share a common structural motif J Biol Chem 2009 284 28674 28681 19692336 10.1074/jbc.M109.036459 PMC2781411

[91] Diep DB Skaugen M Salehian Z Holo H Nes IF Common mechanisms of target cell recognition and immunity for class II bacteriocins Proc Natl Acad Sci USA 2007 104 2384 2389 17284603 10.1073/pnas.0608775104 PMC1892938

[92] Schmidt EW Nelson JT Rasko DA Sudek S Eisen JA Haygood MG Ravel J Patellamide A and C biosynthesis by a microcin-like pathway in Prochloron didemni, the cyanobacterial symbiont of Lissoclinum patella Proc Natl Acad Sci USA 2005 102 7315 7320 15883371 10.1073/pnas.0501424102 PMC1091749

[93] Kiss A Baliko G Csorba A Chuluunbaatar T Medzihradszky KF Alfoldi L Cloning and characterization of the DNA region responsible for Megacin A-216 production in Bacillus megaterium 216 J Bacteriol 2008 190 6448 6457 18689470 10.1128/JB.00557-08 PMC2565993

[94] Pons AM Lanneluc I Cottenceau G Sable S New developments in non-post translationally modified microcins Biochimie 2002 84 531 537 12423797 10.1016/s0300-9084(02)01416-5

[95] Parks WM Bottrill AR Pierrat OA Durrant MC Maxwell A The action of the bacterial toxin, microcin B17, on DNA gyrase Biochimie 2007 89 500 507 17276574 10.1016/j.biochi.2006.12.005

[96] Bieler S Silva F Soto C Belin D Bactericidal activity of both secreted and nonsecreted microcin E492 requires the mannose permease J Bacteriol 2006 188 7049 7061 17015644 10.1128/JB.00688-06 PMC1636244

[97] Bastos MC Ceotto H Coelho ML Nascimento JS Staphylococcal antimicrobial peptides: relevant properties and potential biotechnological applications Curr Pharm Biotechnol 2009 10 38 61 19149589 10.2174/138920109787048580

[98] Bierbaum G Sahl HG Lantibiotics: mode of action, biosynthesis and bioengineering Curr Pharm Biotechnol 2009 10 2 18 19149587 10.2174/138920109787048616

[99] Breukink E A lesson in efficient killing from two-component lantibiotics Mol Microbiol 2006 61 271 273 16771845 10.1111/j.1365-2958.2006.05239.x

[100] Cooper LE McClerren AL Chary A van der Donk WA Structure-activity relationship studies of the two-component lantibiotic haloduracin Chem Biol 2008 15 1035 1045 18940665 10.1016/j.chembiol.2008.07.020 PMC2633096

[101] Lawton EM Ross RP Hill C Cotter PD Two-peptide lantibiotics: a medical perspective Mini-Rev Med Chem 2007 7 1236 1247 18220976 10.2174/138955707782795638

[102] Nissen-Meyer J Rogne P Oppegard C Haugen HS Kristiansen PE Structure-Function Relationships of the Non-Lanthionine-Containing Peptide (class II) Bacteriocins Produced by Gram-Positive Bacteria Curr Pharm Biotechnol 2009 10 19 37 19149588 10.2174/138920109787048661

[103] Draper LA Ross RP Hill C Cotter PD Lantibiotic immunity Curr Protein Pept Sci 2008 9 39 49 18336322 10.2174/138920308783565750

[104] Lubelski J Rink R Khusainov R Moll GN Kuipers OP Biosynthesis, immunity, regulation, mode of action and engineering of the model lantibiotic nisin Cell Mol Life Sci 2008 65 455 476 17965835 10.1007/s00018-007-7171-2 PMC11131864

[105] Kjos M Nes IF Diep DB Class II one-peptide bacteriocins target a phylogenetically defined subgroup of mannose phosphotransferase systems on sensitive cells Microbiology 2009 155 2949 2961 19477899 10.1099/mic.0.030015-0

[106] Romanenko LA Uchino M Kalinovskaya NI Mikhailov VV Isolation, phylogenetic analysis and screening of marine mollusc-associated bacteria for antimicrobial, hemolytic and surface activities Microbiol Res 2008 163 633 644 19216104 10.1016/j.micres.2006.10.001

[107] Wilson GS Raftos DA Corrigan SL Nair SV Diversity and antimicrobial activities of surface-attached marine bacteria from Sydney Harbour, Australia Microbiol Res 2009 in Press 10.1016/j.micres.2009.05.007 19656668

[108] Selvin J Joseph S Asha KR Manjusha WA Sangeetha VS Jayaseema DM Antony MC Denslin Vinitha AJ Antibacterial potential of antagonistic Streptomyces sp. isolated from marine sponge Dendrilla nigra FEMS Microbiol Ecol 2004 50 117 122 19712370 10.1016/j.femsec.2004.06.007

[109] Morris JJÂG Cholera and Other Types of Vibriosis: A Story of Human Pandemics and Oysters on the Half Shell Clin Infect Dis 2003 37 272 280 12856219 10.1086/375600

[110] Zai AS Ahmad S Rasool SA Bacteriocin production by indigenous marine catfish associated *Vibrio* spp Pak J Pharm Sci 2009 22 162 167 19339226

[111] Carraturo A Raieta K Ottaviani D Russo GL Inhibition of *Vibrio parahaemolyticus* by a bacteriocin-like inhibitory substance (BLIS) produced by *Vibrio mediterranei* 1 J Appl Microbiol 2006 101 234 241 16834611 10.1111/j.1365-2672.2006.02909.x

[112] Prasad S Morris PC Hansen R Meaden PG Austin B A novel bacteriocin-like substance (BLIS) from a pathogenic strain of *Vibrio harveyi* Microbiology 2005 151 3051 3058 16151215 10.1099/mic.0.28011-0

[113] Zhang X-H Austin B Pathogenicity of Vibrio harveyi to salmonids J Fish Dis 2000 23 93 102

[114] McCall JO Sizemore RK Description of a bacteriocinogenic plasmid in *Beneckea harveyi* Appl Environ Microbiol 1979 38 974 979 317423 10.1128/aem.38.5.974-979.1979 PMC243617

[115] Hoyt PR Sizemore RK Competitive Dominance by a Bacteriocin-Producing *Vibrio harveyi* Strain Appl Environ Microbiol 1982 44 653 658 16346094 10.1128/aem.44.3.653-658.1982 PMC242072

[116] Shehane SD Sizemore RK Isolation and preliminary characterization of bacteriocins produced by *Vibrio vulnificus* J Appl Microbiol 2002 92 322 328 11849361 10.1046/j.1365-2672.2002.01533.x

[117] Sugita H Matsuo N Hirose Y Iwato M Deguchi Y Vibrio sp. strain NM 10, isolated from the intestine of a Japanese coastal fish, has an inhibitory effect against Pasteurella piscicida Appl Environ Microbiol 1997 63 4986 4989 9406423 10.1128/aem.63.12.4986-4989.1997 PMC168829

[118] Pedron Moro EM Niederauer Weiss RD Salete Friedrich R Paiva Nunes M Bacteriocin-like Substance of *Aeromonas hydrophila* Mem Inst Oswaldo Cruz 1997 92 115 116 10.1590/s0074-02761997000100024 9302421

[119] Messi P Guerrieri E Bondi M Bacteriocin-like substance (BLS) production in *Aeromonas hydrophila* water isolates FEMS Microbiol Lett 2003 220 121 125 12644237 10.1016/S0378-1097(03)00092-2

[120] Pirzada ZA Ali SA Khan BM Rasool SA Production And Physico-Chemical Characterization Of Bacteriocins-Like Inhibitory Substances From Marine Bacterium ZM81 Pak J Biol Sci 2004 7 2026 2030

[121] Longeon A Peduzzi J Barthelemy M Corre S Nicolas J-L Guyot M Purification and Partial Identification of Novel Antimicrobial Protein from Marine Bacterium *Pseudoalteromonas* Species Strain X153 Mar Biotechnol 2004 6 633 641 15747093 10.1007/s10126-004-3009-1

[122] Ringø E Gatesoupe FJ Lactic acid bacteria in fish: a review Aquaculture 1998 160 177 203

[123] Rihakova J Belguesmia Y Petit VW Pilet MF Prevost H Dousset X Drider D Divercin V41 from gene characterization to food applications: 1998–2008, a decade of solved and unsolved questions Lett Appl Microbiol 2009 48 1 7 19018960 10.1111/j.1472-765X.2008.02490.x

[124] Hosseini SV Arlindo S Böhme K Fernández-No C Calo-Mata P Barros-Velázquez J Molecular and probiotic characterization of bacteriocin-producing Enterococcus faecium strains isolated from non fermented animal foods J Appl Microbiol 2009 107 1392 1403 19426265 10.1111/j.1365-2672.2009.04327.x

[125] Pinto AL Fernandes M Pinto C Albano H Castilho F Teixeira P Gibbs PA Characterization of anti-Listeria bacteriocins isolated from shellfish: Potential antimicrobials to control non-fermented seafood Int J Food Microbiol 2009 129 50 58 19081155 10.1016/j.ijfoodmicro.2008.11.005

[126] Duffes F Leroi F Boyaval P Dousset X Inhibition of *Listeria monocytogenes* by *Carnobacterium* spp. strains in a simulated cold smoked fish system stored at 4 °C Int J Food Microbiol 1999 47 33 42 10357271 10.1016/s0168-1605(98)00206-2

[127] Métivier A Pilet M-F Dousset X Sorokine O Anglade P Zagorec M Piard J-C Marion D Cenatiempo Y Fremaux C Divercin V41, a new bacteriocin with two disulphide bonds produced by *Carnobacterium divergens* V41: primary structure and genomic organization Microbiology 1998 144 2837 2844 9802025 10.1099/00221287-144-10-2837

[128] Pilet M-F Dousser X Barre R Novel G Dezmazeaud M Piard J-C Evidence for two bacteriocins produced by *Carnobacterium piscicola* and *Carnobacterium divergens* isolated from fish and active against *Listeria monocytogenes* J Food Prot 1995 58 256 262 10.4315/0362-028X-58.3.256 31137289

[129] Richard C Drider D Elmorjani K Marion D Prevost H Heterologous Expression and Purification of Active Divercin V41, a Class IIa Bacteriocin Encoded by a Synthetic Gene in *Escherichia coli* J Bacteriol 2004 186 4276 4284 15205430 10.1128/JB.186.13.4276-4284.2004 PMC421597

[130] Bhugaloo-Vial P Dousset X Metivier A Sorokine O Anglade P Boyaval P Marion D Purification and amino acid sequences of piscicocins V1a and V1b, two class IIa bacteriocins secreted by *Carnobacterium piscicola* V1 that display significantly different levels of specific inhibitory activity Appl Environ Microbiol 1996 62 4410 4416 8953713 10.1128/aem.62.12.4410-4416.1996 PMC168268

[131] Moore BS Biosynthesis of marine natural products: microorganisms (Part A) Nat Prod Rep 2005 22 580 593 16193157 10.1039/b404737k

[132] Sudek S Haygood MG Youssef DTA Schmidt EW Structure of Trichamide, a Cyclic Peptide from the Bloom-Forming Cyanobacterium *Trichodesmium erythraeum*, Predicted from the Genome Sequence Appl Environ Microbiol 2006 72 4382 4387 16751554 10.1128/AEM.00380-06 PMC1489667

[133] Ziemert N Ishida K Quillardet P Bouchier C Hertweck C de Marsac NT Dittmann E Microcyclamide Biosynthesis in Two Strains of *Microcystis aeruginosa*: from Structure to Genes and Vice Versa Appl Environ Microbiol 2008 74 1791 1797 18245249 10.1128/AEM.02392-07 PMC2268316

[134] Long PF Dunlap WC Battershill CN Jaspars M Shotgun Cloning and Heterologous Expression of the Patellamide Gene Cluster as a Strategy to Achieving Sustained Metabolite Production13 Chem Bio Chem 2005 6 1760 1765 10.1002/cbic.200500210 15988766

[135] Jüttner F Todorova AK Walch N von Philipsborn W Nostocyclamide M: a cyanobacterial cyclic peptide with allelopathic activity from Nostoc 31 Phytochemistry 2001 57 613 619 11394868 10.1016/s0031-9422(00)00470-2

[136] Banker R Carmeli S Tenuecyclamides A-D, Cyclic Hexapeptides from the Cyanobacterium Nostoc spongiaeforme var. tenue J Nat Prod 1998 61 1248 1251 9784161 10.1021/np980138j

[137] Linington RG Gonzalez J Urena L-D Romero LI Ortega-Barria E Gerwick WH Venturamides A and B: Antimalarial Constituents of the Panamanian Marine *Cyanobacterium Oscillatoria* sp J Nat Prod 2007 70 397 401 17328572 10.1021/np0605790

[138] Ogino J Moore RE Patterson GML Smith CD Dendroamides, New Cyclic Hexapeptides from a Blue-Green Alga. Multidrug-Resistance Reversing Activity of Dendroamide A J Nat Prod 1996 59 581 586 8786364 10.1021/np960178s

[139] Ishida K Nakagawa H Murakami M Microcyclamide, a Cytotoxic Cyclic Hexapeptide from the Cyanobacterium *Microcystis aeruginosa* J Nat Prod 2000 63 1315 1317 11000050 10.1021/np000159p

[140] Gatesoupe FJ Updating the importance of lactic acid bacteria in fish farming: natural occurrence and probiotic treatments J Mol Microbiol Biotechnol 2008 14 107 114 17957117 10.1159/000106089

[141] Wang Y-B Li J-R Lin J Probiotics in aquaculture: Challenges and outlook Aquaculture 2008 281 1 4

[142] Hong HA Duc LH Cutting SM The use of bacterial spore formers as probiotics FEMS Microbiol Rev 2005 29 813 835 16102604 10.1016/j.femsre.2004.12.001

[143] Vine NG Leukes WD Kaiser H Probiotics in marine larviculture FEMS Microbiol Rev 2006 30 404 427 16594964 10.1111/j.1574-6976.2006.00017.x

[144] Das S Ward L Burke C Prospects of using marine actinobacteria as probiotics in aquaculture Appl Microbiol Biotechnol 2008 81 419 429 18841358 10.1007/s00253-008-1731-8

[145] von Wright A Regulating the Safety of Probiotics - The European Approach Curr Pharm Des 2005 11 17 23 15638749 10.2174/1381612053382322

[146] 101.70, C. Subpart E-Specific Requirements for Health Claims Code Fed Regul 2005 21 126 129

[147] Sahu M Swarnakumar N Sivakumar K Thangaradjou T Kannan L Probiotics in aquaculture: importance and future perspectives Indian J Microbiol 2008 48 299 308 10.1007/s12088-008-0024-3 PMC3476780 23100726

[148] Guo J-J Liu K-F Cheng S-H Chang CI Lay J-J Hsu Y-O Yang J-Y Chen T-I Selection of probiotic bacteria for use in shrimp larviculture Aquaculture Res 2009 40 609 618

[149] Ruiz-Ponte C Samain JF Sánchez JL Nicolas JL The Benefit of a Roseobacter Species on the Survival of Scallop Larvae Mar Biotechnol 1999 1 52 59 10373610 10.1007/pl00011751

